# Electron Spin Resonance at the Single‐Molecule Scale

**DOI:** 10.1002/anie.202506539

**Published:** 2025-08-19

**Authors:** Lisanne Sellies, Jascha Repp

**Affiliations:** ^1^ IBM Research Europe – Zurich Säumerstrasse 4 Rüschlikon 8803 Switzerland; ^2^ Institute of Experimental and Applied Physics and Regensburg Center for Ultrafast Nanoscopy University of Regensburg Regensburg Germany

**Keywords:** NV centers, Optically detected magnetic resonance, Scanning probe microscopy, Single‐molecule studies

## Abstract

Electron spin resonance (ESR) is a widely employed spectroscopic technique for studying systems with unpaired electron spins, such as molecular radicals. Typically, many billions of spins are required to get a detectable ESR signal, which is subject to extensive ensemble averaging. Downscaling ESR to a single molecule allows studying the signatures of each individual molecule separately, applicable to biomolecules in their native environment, for example. Single‐molecule ESR offers several novel research avenues, such as in quantum sensing with a single molecule. Over the last decades, four different single‐molecule ESR approaches have been developed, which rely on either optically detected magnetic resonance or scanning‐probe microscopy. An introduction into these four approaches including their deployment in pioneering works will be provided.

## Graphical abstract

This minireview provides an overview of the four electron‐spin‐resonance techniques with single‐molecule sensitivity, relying on either optically detected magnetic resonance or scanning‐probe microscopy. After introducing underlying common concepts, it reviews the working principles, experimental conditions and key achievements.

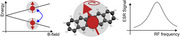

## 1 Introduction

Electron spin resonance (ESR) – also called electron paramagnetic resonance (EPR) – is a widely applied technique in analytical chemistry. It is a magnetic‐resonance technique, meaning that quantum transitions of magnetic moments are resonantly driven and detected. In ESR these transitions are between electronic spin states, in contrast to nuclear spin states in the well‐known magnetic‐resonance technique nuclear magnetic resonance (NMR).

Conventional ESR can be used for determination of the structure of large molecules, the dynamics of spin centers and the spatial distribution of electron spins. The applications range from defect characterization in crystal structures to structure determination of large biomolecules to medicine.^[^ ^1^ ^]^ Because quantum systems can be coherently driven with ESR, its application to spin‐based quantum computing is also envisioned.^[^ ^2^ ^]^ Hence, ESR is relevant to research fields as diverse as analytical chemistry, bio‐imaging, medicine, and quantum information technology.

But what are the potential benefits of pursuing ESR at the single‐molecule scale? The most obvious one is that ensemble averaging is avoided when detecting a single molecule. Ensemble averaging leads to a broadening of spectra, such that some subtle but important molecule‐intrinsic details may get hidden in the inhomogeneous broadening. Further, the environment differs from molecule to molecule such that the interaction with the environment is difficult to quantify in an ensemble average. In addition, single‐molecule ESR‐based imaging would allow imaging and following individual tagged molecules. ESR on single molecules could also be used as a probe in so‐called quantum sensing applications,^[^ ^3^ ^]^ in which the object of interest is not the single molecule itself, but the spins in its environment. For such an application, single‐spin detection is crucial, since the resulting signal should stem from only one local probe. Finally, the realization of an universal quantum computer requires the control of entanglement between individual q‐bits, which is difficult to realize on an ensemble average.^[^ ^4^, ^5^ ^]^

## 2 General Concepts of ESR

Conventional ESR relies on the absorption of electromagnetic radiation at radio frequency (RF) that drives the spin transitions, as first experimentally demonstrated in 1945 by Zavoisky.^[^ ^6^ ^]^ To detect the absorption of electromagnetic radiation a certain amount of the substance is required. Typically, this minimum amounts to 10^9^ to 10^14^ molecules, whereas in dedicated and very sophisticated experiments a detection of few tens of molecules could be reached.^[^ ^7^ ^]^ Before discussing how single‐spin sensitivity can be achieved, we will first review general principles of ESR.

### 2.1 ESR Working Principle

**Figure 1:**
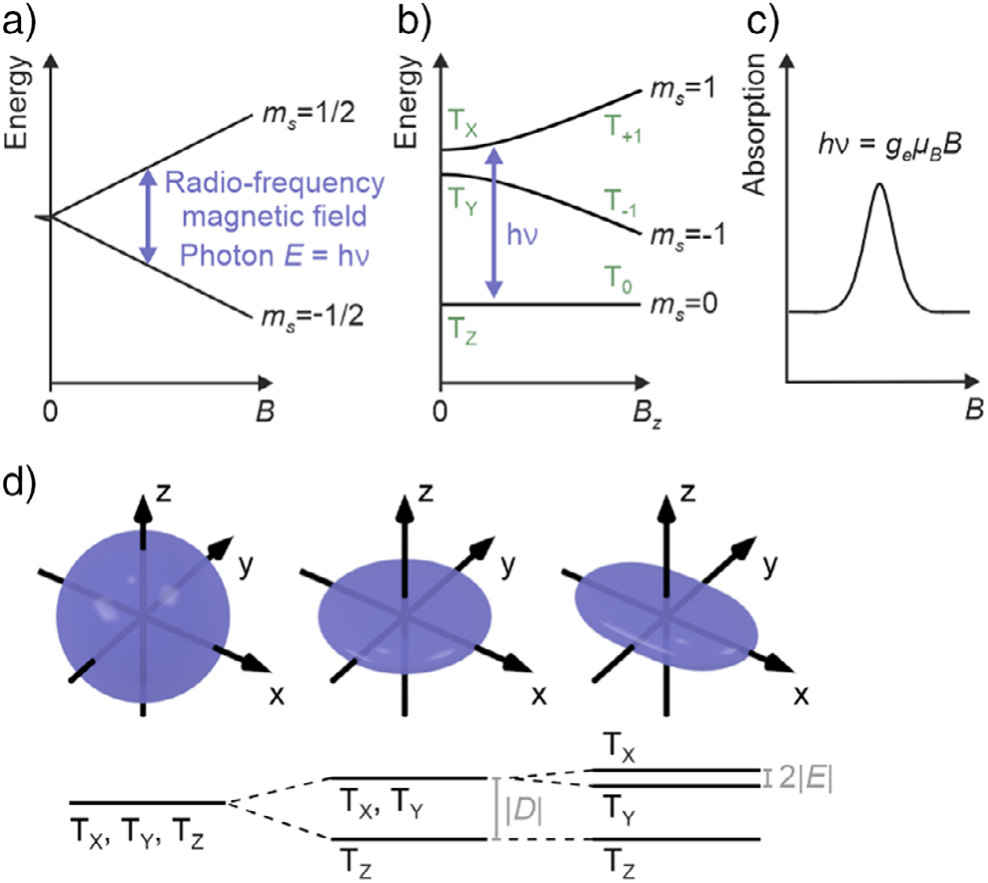
a), b) Energy diagrams in ESR. An RF field can drive transitions between spin states if its photon energy hν matches the energy splitting between the spin states, depicted for Zeeman‐split spin states of an *S* = ½ a) and an *S *= 1 b) system in an external static magnetic field *B*. Although the field's direction with respect to the molecular orientation matters, the schematic depicts only one exemplary field direction. c) A conventional ESR spectrum is measured by sweeping *B* at a fixed frequency (or vice versa). At resonance the RF radiation is absorbed (note that in conventional continuous‐wave ESR the signal detection is differential and signals appear as derivatives of the resonance curve). d) Illustration of the zero‐field splitting due to the dipole‐dipole coupling of two unpaired electron spins. Because of the anisotropy of the dipole‐dipole interaction, the alignment of the spins with respect to the spatial directions matters. Whereas for a spherical density distribution of the interacting dipoles, the degeneracy of the three triplet sublevels is preserved at *B *= 0, it is sequentially lifted for cylindrical and other density distributions of lower symmetry, as indicated. The zero‐field splitting is typically quantified by the parameters *D* and *E*. Panel d adapted from Ref. [16]; license CC‐BY 4.0.

As mentioned above, central to ESR is the resonant driving of a quantum transition between two energy levels. This requires i) having (at least) two spin states at different energies, usually achieved by application of a static magnetic field ***B*** and ii) being able to drive the transition between them as mentioned above, see Figure 1a. However, the driving RF field acts equally in both directions, that is, driving the system from level |0⟩ to |1⟩ has the same rate as the reverse transition from |1⟩ to |0⟩. Consequently, it requires iii) a different initial population in |0⟩ and |1⟩ to have a net effect of the driving. This difference in initial population can be either given by thermal equilibrium or the spin system can be prepared out of equilibrium. Finally, iv) a detection principle is needed that translates the RF‐induced population transfer into a detectable signal. As mentioned above, in conventional ESR this detection principle relies on measuring the absorption, see Figure 1c. The above considerations define four requirements i)–iv) for ESR, which are met differently in the various techniques. For example, the sensing in single‐molecule ESR differs from detecting the absorption. For techniques that rely on other detection mechanisms than absorption, it is often name‐defining, as, e.g., in optically detected magnetic resonance (ODMR)^[^ ^8^, ^9^, ^10^ ^]^ or electronically detected magnetic resonance (EDMR).^[^ ^11^, ^12^, ^13^, ^14^, ^15^ ^]^

### 2.2 ESR Energy Level Scheme

The level scheme of the spin manifold is most important for ESR, as it defines the non‐degenerate states on which ESR is conducted and determines the resonant condition. The most important terms defining the level scheme are the Zeeman interaction with an external magnetic field, the exchange interaction, the dipole‐dipole interaction and various hyperfine interaction terms with nuclear spins. Among these, the Zeeman energy and the dipole‐dipole interaction between electrons within a single molecule have the right magnitude to lead to energy differences that can conveniently be probed by ESR, whereas the exchange interaction between electrons within a single molecule has a much larger magnitude. The remaining interactions determine the more detailed structure or allow the coupling of the spin system to the atomic‐scale neighborhood. Consequently, for spin‐doublet systems with one unpaired spin ESR requires a static external magnetic field ***B***, which splits the two spin states with magnetic quantum numbers *m*_*S*_ = −1/2 and *m*_*S*_ = 1/2 according to the Zeeman term of the Hamiltonian *H*_Z_ = *gμ*_B_ ***S*** · ***B***, with the Landé factor *g*, the Bohr magneton *μ*_B_, the electron spin operator ***S*** and the magnetic field ***B*** (see Figure 1a).

Molecules in triplet states (or higher multiplicity) exhibit an intrinsic zero‐field splitting of these states (see Figure 1b), which is mostly due to the magnetic dipole‐dipole interaction.^[^ ^17^ ^]^ The latter is highly anisotropic, that is, directionally dependent. In other words, for given spin orientations, the dipole‐dipole interaction strongly differs and even changes sign for different relative positions of the two spins. The distribution of spatial positions of the electron spins is given by the orbital densities of the two interacting unpaired electrons, the confinement of which is, for a typical molecule, different along the three molecular axes. The anisotropy of the dipole‐dipole interaction together with the non‐uniformity of the orbital densities give rise to an energy difference in the range of microelectronvolts for the three spin sublevels, see Figure 1d. Since the density distribution of the spins is decisive for the zero‐field splitting, the latter is a fingerprint of the orbital densities and thereby of the molecular species. For the same reason, the quantization axes of the triplet sublevels at zero field are tied to the molecule's real‐space directions. The zero‐field splitting can be cast into the Hamiltonian as *H_ZF_* = ***SDS*** with the dipole‐dipole‐interaction tensor ***D***, which in turn can be parameterized using two values, namely the axial (*D*) and rhombic (*E*) component. It enables performing ESR even without external magnetic field.

**Figure 2:**
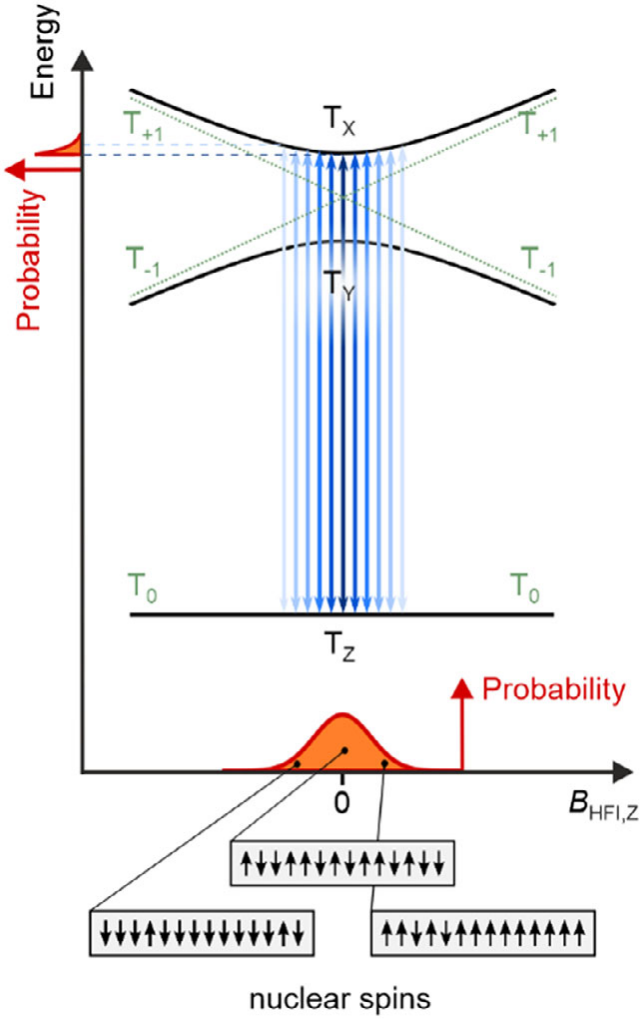
Schematic to explain the asymmetric line shape resulting from hyperfine interaction with multiple nuclear spins. The hyperfine interaction can be considered as an effective magnetic field *B*_HFI_ (considered here along the *z* direction) acting on the electron spins and thereby affecting the triplet states’ energies. The fluctuating nuclear spin configurations give rise to a range of energies and therefore RF transitions (blue arrows) that satisfy the resonance condition. Weighted with the corresponding probability distribution this results in an asymmetric line shape (top left). Adapted from Ref. [16]; license CC‐BY 4.0.

Hyperfine interactions, that is, the hyperfine coupling of the nuclear spins to the electron spins, have a subtle influence on the level scheme, which thereby entails information about the nuclear spin system of the molecule. The hyperfine interaction contributes only to second order to ESR spectra measured at zero‐magnetic field. As illustrated in Figure 2, an asymmetric line shape results from the hyperfine interaction of multiple nuclear spins.^[^ ^18^ ^]^

Since hyperfine interactions couple the nuclear spins to the electron spin system, they also allow – roughly speaking – the use of ESR to perform nuclear magnetic resonance, a technique called electron nuclear double resonance (ENDOR).

### 2.3 Coherence and Bloch‐Sphere Representation

A spin‐manipulation process in ESR includes aspects of quantum mechanical coherence as well as decoherence. On the one side, the spin manipulation itself is a coherent manipulation of the quantum state. On the other side, dephasing of the individual spins leads to decoherence in an ensemble. Although there is no ensemble of spins in single‐spin experiments, the experimental signal typically represents an average over many individual spin manipulations being conducted subsequently. Hence, the result represents a repeated‐experiment ensemble, such that a theoretical description is analogous to an ensemble of particles. Since the theoretical description proceeds analogous to conventional ESR, we refer to the existing literature.^[^ ^19^, ^20^ ^]^

**Figure 3:**
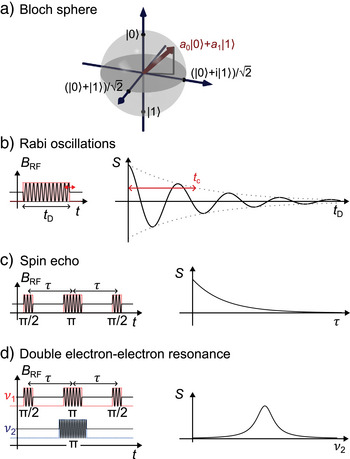
a) Representation of a quantum state of an effective two‐level system (levels |0⟩ and |1⟩) as a Bloch vector (red) on a Bloch sphere. A few exemplary states are indicated. b) Resonant driving leads to the population oscillating between the two levels as a function of pulse duration *t*_D_, called Rabi oscillations. The falling envelope function of the signal *S* reflects the decay of spin polarization. c) To measure dephasing of the spin system, spin echo experiments are used, in which part of the dephasing occurring during an initial phase is refocused in a second phase. Depending on the experimental conditions, the last π/2 pulse is either necessary or omitted. d) In double‐resonance experiments, pulses at different carrier frequencies are superimposed, each being at resonance with a different spin, such as an observer (1) and a pump (or drive) (2) spin. Many different pulse schemes exist for such experiments.

The Bloch sphere is a very useful way to represent a quantum state of a two‐level system, including coherence and decoherence. In this representation – ideally suitable for ESR – any pure state is represented by a Bloch vector ending on the surface of the Bloch sphere, with the two eigenstates |0⟩ and |1⟩ being located at the two poles, as illustrated in Figure 3a. The polar and azimuthal angles encode the contributions of |0⟩ and |1⟩ to the quantum state Ψ including their phase relation. A coherent manipulation of Ψ is therefore characterized by a change in these angles, to which is being referred in the denotation of π/2‐ or π‐pulses in pulsed ESR. Decoherence can be represented by a Bloch vector being shorter than the radius of the sphere: the vector designates only the coherent part of the population of the system.

The decay of spin polarization occurs in two distinct ways: The eigenstates decay into other eigenstates of the system with characteristic lifetimes. This process restores the Boltzmann distribution with a relaxation time *T*_1_. Independently, a coherent superposition of two eigenstates has a certain relative phase of the amplitudes of states |0⟩ and |1⟩. In an ensemble, the phase of the individual samples may lose coherence with a relaxation time *T*_2_. Whereas – strictly speaking – *T_1_* and *T_2_* denote the respective relaxation times, they are commonly used for the underlying processes as well. Resulting from the construction of the Bloch sphere, *T*_1_ affects the vertical and *T*_2_ the horizontal components of the Bloch vector, respectively.

### 2.4 Pulsed ESR

By applying the RF driving as pulses, coherence can be exploited to single out specific electron‐spin interactions and for the extraction of the corresponding relaxation times. The key concepts relevant to single‐molecule ESR are described next.

As stated above, the RF field coherently drives the population between two quantum states. Starting with a population imbalance and driving the transition for only a short pulse duration, enables the observation of the population oscillating back and forth between the two states. These so‐called Rabi oscillations can be measured by varying the duration of the RF pulse, see Figure 3b. Rabi oscillations can be used as a rough measure for the coherence time. However, the RF field itself can have a strong influence on the coherence time. Consequently, the decay of the oscillations depends on the power of the RF pulse used.^[^ ^21^, ^22^ ^]^

The relaxation of the transversal component of the spin, *T*_2_ relaxation, can be measured accurately with a two‐pulse experiment called Hahn or spin echo, as illustrated in Figure 3c. Upon applying a π/2 pulse, the Bloch vector will be driven into the transversal plane. During the subsequent evolution time *τ*_1_ the spin will precess according to its Lamor frequency. This Larmor frequency depends on the configuration of the spins in the environment, such as the nuclear spins. Different molecules (or the same molecule in different time instances) differ in their Larmor frequency and will consequently dephase. Then, a π pulse is applied to flip the Bloch vector into the transversal plane. During a final evolution time *τ*_2_, the spin precession is reversed, which allows the spins to rephase if *τ *= *τ*_1_ = *τ*_2_, resulting in an echo signal. The echo can be detected as a change in population by a final π/2 pulse. If a *T*_2_ relaxation process happens during one of the evolution times, it will change the Larmor frequency and thereby reduce the measured echo signal. The *T*_2_ relaxation constant can be obtained by varying the duration of the evolution periods *τ*.

Pulsed ESR can also be used to directly measure the spins that are coupled to the spins that are being measured. To this end, a double electron‐electron resonance (DEER) pulse sequence can be used. One implementation of such a pulse sequence is shown in Figure 3d. It consists of a spin echo applied to the sensor spin and a π pulse applied to the coupled spin, simultaneously with the π pulse applied to the sensor spin. This spin echo refocuses the dephasing that is due to electron and nuclear spins in the environment, as explained above. Because of the pulse applied to the coupled spin, the dephasing due to the coupled spin is not refocused, which will affect the phase of the sensor spin. This can be detected as a difference in the sensor's spin population by a final π/2 pulse applied to the sensor spin. Sweeping the frequency of the pulse on the coupled spin allows measuring its ESR signal via the sensor spin. DEER pulse sequences can also be used for the quantification of the coupling between the sensor spin and the coupled spin.

## 3 Overview of Single‐Molecule ESR

The most important requirement for single‐molecule ESR is the ability to sense the signal arising from a single spin. Single‐spin systems can be realized not only in molecular systems but also in quantum dots or individual atoms. In fact, single‐spin sensitivity has been developed in many experiments that did not necessarily address individual molecules^[^ ^11^, ^12^, ^13^, ^14^, ^15^, ^18^, ^23^, ^24^, ^25^, ^26^ ^]^ and will not be reviewed here. Most importantly, for single‐spin sensitivity a fundamentally different way of detection is required, namely, by mapping the spin transition onto a property that can be more sensitively detected.

ODMR proceeds via luminescence, for which cross sections are several orders of magnitude larger than for microwave absorption,^[^ ^27^ ^]^ enabling single‐spin and also single‐molecule sensitivity. ODMR is therefore one of the techniques discussed here. In addition, by means of ODMR, nitrogen‐vacancy (NV) centers in diamond can be used to sense individual molecules. In addition, two scanning‐probe based ESR approaches exist, which rely on sensing either the spin polarization itself (with spin‐polarized scanning tunneling microscopy (STM)) or single‐electron charges (with atomic force microscopy (AFM)). While in ESR‐STM and NV‐center‐based ODMR, molecules with a single unpaired electron spin were studied, in ESR‐AFM and single‐molecule ODMR, molecules in their spin‐1 triplet state were studied. In the latter techniques, typically no static magnetic field was applied. ESR at zero‐magnetic field on spins in their triplet state is called zero‐field triplet state ESR.^[^ ^17^ ^]^ Table 1 provides an overview of the existing single‐molecule ESR techniques, comparing key aspects and parameters.

**Table 1:** Overview of the existing single‐molecule ESR techniques.

	ODMR	NV centers	ESR‐STM	ESR‐AFM
Spin state studied	Triplet excited state	Doublet ground state (using the NV triplet ground state)	Doublet ground state	Triplet excited state
Key achievements	First ESR and pulsed ESR on single molecules Distinguishing individual isotopomers	Room‐temperature single‐molecule detection Single‐molecule dynamics	Sub‐Å MRI Single‐molecule ESR‐based quantum sensor	Locating individual isotopomers Long coherence times
Dominant splitting mechanism	Zero‐field	Zeeman (molecule)	Zeeman	Zero‐field
Population difference given by	Optical spin pumping^a)^	Optical spin pumping (NV)^a)^	Thermally or spin‐transfer torque	Triplet lifetimes
*B*‐field range^b)^	0 – 10 mT	10 – 100 mT	Up to 2.5 T	0 T
Temperature^b)^	1.5 – 1.8 K	Room temperature	0.4 – 4.5 K	8 K
RF field^b)^	Single‐loop coil	Waveguide	Electric field combined with magnetic tip	Stripline
Detection	Fluorescence	Fluorescence	Magneto‐resistive tunneling	Spin‐to‐charge conversion and force

^a)^ Refers to optical pumping followed by spin‐dependent relaxation (and not to selective driving of specific spin transitions by circularly polarized light).

^b)^ The entries refer to the experimentally explored parameter space for single‐molecule measurements and not to fundamental physical boundaries.

## 4 Optically‐Detected Magnetic Resonance

### 4.1 Single‐Molecule Fluorescence

ODMR relies on detecting ESR signals via fluorescence. Thus, a prerequisite for ODMR on single molecules is the optical excitation and detection of a single molecule, as was demonstrated by Moerner et al.^[^ ^28^ ^]^ and Orrit et al.^[^ ^29^ ^]^ They did measurements on pentacene molecules embedded in a p‐terphenyl host crystal. Pentacene was optically excited from the singlet ground state S_0_ to the first excited singlet state S_1_. Since every molecule in a host crystal experiences a slightly different environment due to defects in the crystal, this S_0_ → S_1_ transition is inhomogeneously broadened.^[^ ^18^, ^29^ ^]^ At liquid‐helium temperatures, the homogenous line width of an individual molecule is much narrower than the inhomogeneous line width of an ensemble. A single molecule can then be optically addressed by combining excitation in the wing of the inhomogeneously broadened S_0_ → S_1_ transition with a narrow laser focus (approximately 5 µm)^[^ ^18^, ^29^ ^]^ on a thin crystal flake (typical thickness of 10 µm)^[^ ^27^ ^]^ with a low concentration of embedded molecules (10^−7^–10^−9^ mol mol^−1^).^[^ ^27^ ^]^ After the optical excitation, the emitted photon from the S_1_ → S_0_ transition of this molecule can be detected. This luminescence cycle, S_0_ → S_1_ and S_1_ → S_0_, occurs repeatedly, and the fluorescence intensity averaged over many cycles is detected.

### 4.2 Single‐Molecule ODMR

**Figure 4:**
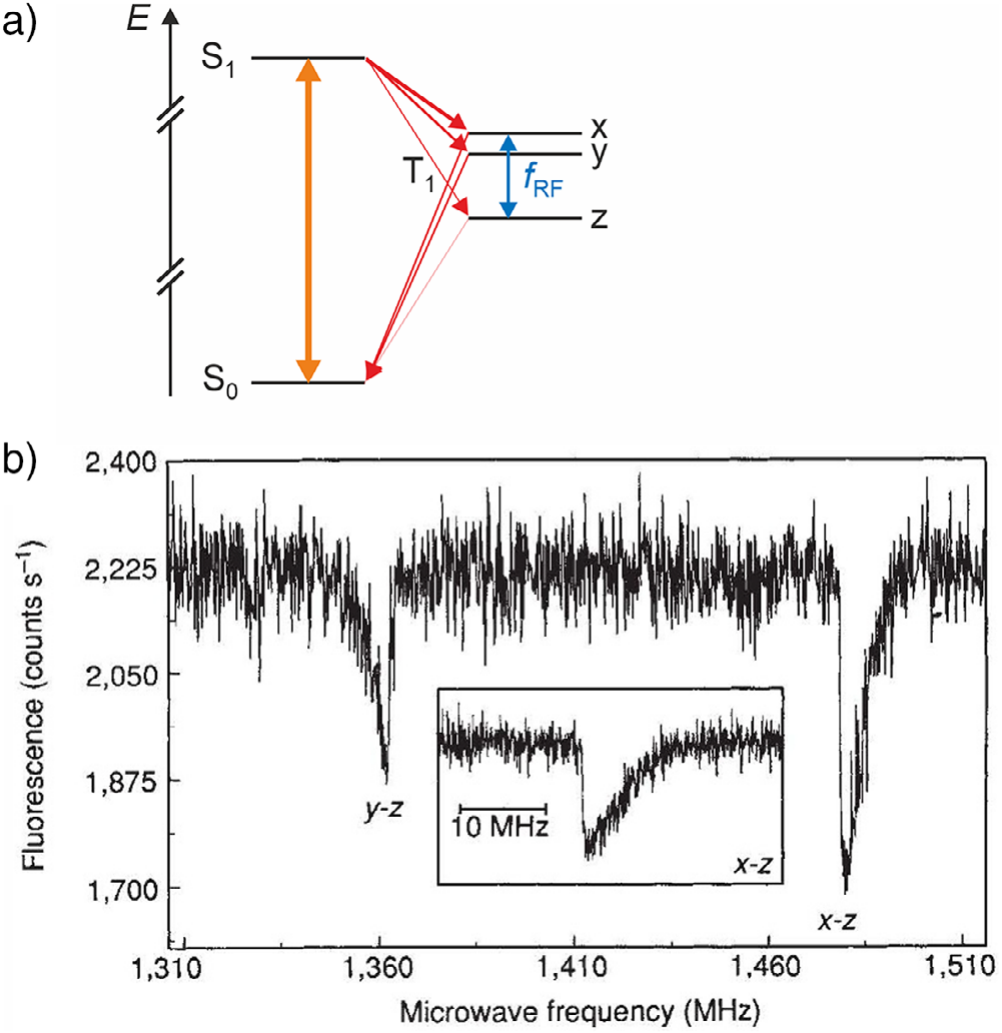
a) Energy‐level scheme of pentacene in zero magnetic field showing the transitions occurring during an ODMR measurement. The orange double‐headed arrow indicates the optical excitation and luminescence, the red arrows the non‐radiative intersystem crossing and the blue arrow the RF driving. Note that, higher‐lying triplet state(s) can be involved but are not shown.^[^ ^31^ ^]^ Based on a figure from Ref. [32]. b) ODMR spectrum of a single pentacene molecule, showing the ESR signals corresponding to two transitions between triplet states as indicated. Reproduced with permission from Ref. [30].

Köhler et al.^[^ ^18^ ^]^ and Wrachtrup et al.^[^ ^30^ ^]^ used this fluorescence detection of a single pentacene molecule to perform zero‐field triplet state ESR. In case of pentacene, there is a significant probability to populate the dark triplet T_1_ state via intersystem crossing (ISC) from S_1_, see Figure 4a. The ISC rates into the three triplet sublevels are different, creating a population difference between the zero‐field‐split sublevels (requirement i and iii), which is out of thermal equilibrium and does not rely on low temperatures (however, single‐molecule fluorescence does). ESR transitions between the optically populated triplet sublevels can be driven by means of an RF magnetic field generated with a single‐loop coil (requirement ii). Since the three triplet sublevels have different lifetimes, driving ESR transitions will modify the overall triplet lifetime, and thereby the average dark‐state population.^[^ ^18^, ^30^ ^]^ This, in turn, changes the fluorescence intensity, which is what is detected (requirement iv). A resulting ESR spectrum for a single pentacene molecule is shown in Figure 4b.

Based on this method applied to pentacene molecules, numerous fascinating experimental achievements have been accomplished at the single molecule level.

### 4.3 Hyperfine Interaction due to a Single Nucleus

The asymmetric line shapes in the ODMR spectra of single pentacene molecules (Figure 4b) are a result of the hyperfine coupling of the electron spins to the fourteen proton spins, as illustrated in Figure 2. Since the hyperfine interaction depends on the nuclear identity, different isotopomers can be discriminated by their spectra: molecules containing a single ^13^C in protonated^[^ ^33^ ^]^ and deuterated^[^ ^34^ ^]^ pentacene and a single ^1^H in an otherwise fully deuterated pentacene molecule^[^ ^32^ ^]^ could be identified.

For such molecules, for which one nucleus dominates the hyperfine splitting, the latter could be resolved by measuring ODMR spectra with a small applied magnetic field (up to 10 mT). The assignment to the respective isotopomers is based on a combination of characteristics of the optical luminescence (ODMR is based on) and ODMR spectra, including shifts, intensities and broadening, in comparison to calculations.^[^ ^32^, ^35^ ^]^ ODMR of different isotopomers could also shed light onto the electron spin density, since for planar aromatic molecules the hyperfine interaction is in good approximation proportional to the spin density at the carbon atom where the ^13^C nucleus is or to which the ^1^H nucleus is bound,^[^ ^35^, ^36^ ^]^ respectively.

### 4.4 Pulsed Single‐Molecule ODMR

Wrachtrup et al. measured Rabi oscillations for a single pentacene molecule in pulsed ODMR.^[^ ^37^ ^]^ The oscillations decay with a decay constant of around 5 µs. They attributed this decay to a fluctuating nuclear spin configuration for every measurement cycle. These nuclear spin fluctuations also gave rise to the asymmetric ESR line shape reflecting all different nuclear spin configurations. Because of these fluctuations, the resonance frequency differed slightly for every individual measurement cycle. Since the Rabi frequency depends on the resonance frequency with respect to the fixed driving frequency, the Rabi frequency will also differ from cycle to cycle, leading to dephasing when averaging over the fluctuating Rabi frequency.^[^ ^38^ ^]^

In addition, they measured a Hahn echo for a single pentacene molecule.^[^ ^39^ ^]^ Analogously to the case above, the repeated measurements of one single molecule represent the temporal ensemble average, allowing a Hahn echo to be measured for a single molecule. The decay of the Hahn echo was attributed to nuclear spin flipping in the embedding matrix. The decay time varied by a factor three for different individual molecules, between 3 and 8 µs. They attributed this variation to a 10% variation in the lattice constants, changing the internuclear dipole‐dipole coupling and thereby the nuclear flip–flop (the simultaneous flipping of two spins in opposing directions conserving the total spin) rate.^[^ ^39^ ^]^

Finally, they used an ENDOR experiment to detect the NMR signals of individual hydrogen nuclei in pentacene‐d_12_h_2_.^[^ ^40^ ^]^ This experiment monitored the change in ESR signal while the radio frequency field was swept through the resonance of an NMR transition.

### 4.5 Discrete Blinking Detection

The only molecule that has been measured with the above‐described single‐molecule ODMR approach is pentacene. This can be attributed to the stringent conditions that need to be fulfilled. On the one hand, the molecule should be suitable for optical single‐molecule spectroscopy, which requires good photophysical and photochemical stability as well as a large singlet emission rate. A large singlet emission rate is obtained if the fluorescence quantum yield is large, the intersystem‐crossing probability into the triplet state is small and the triplet and singlet lifetimes are short.^[^ ^27^ ^]^ On the other hand, ODMR requires different depopulation rates and a different ratio of population and depopulation rates for the three triplet sublevels. While these conditions admit gradations, only a few compounds satisfy them to a practicable degree.

**Figure 5:**
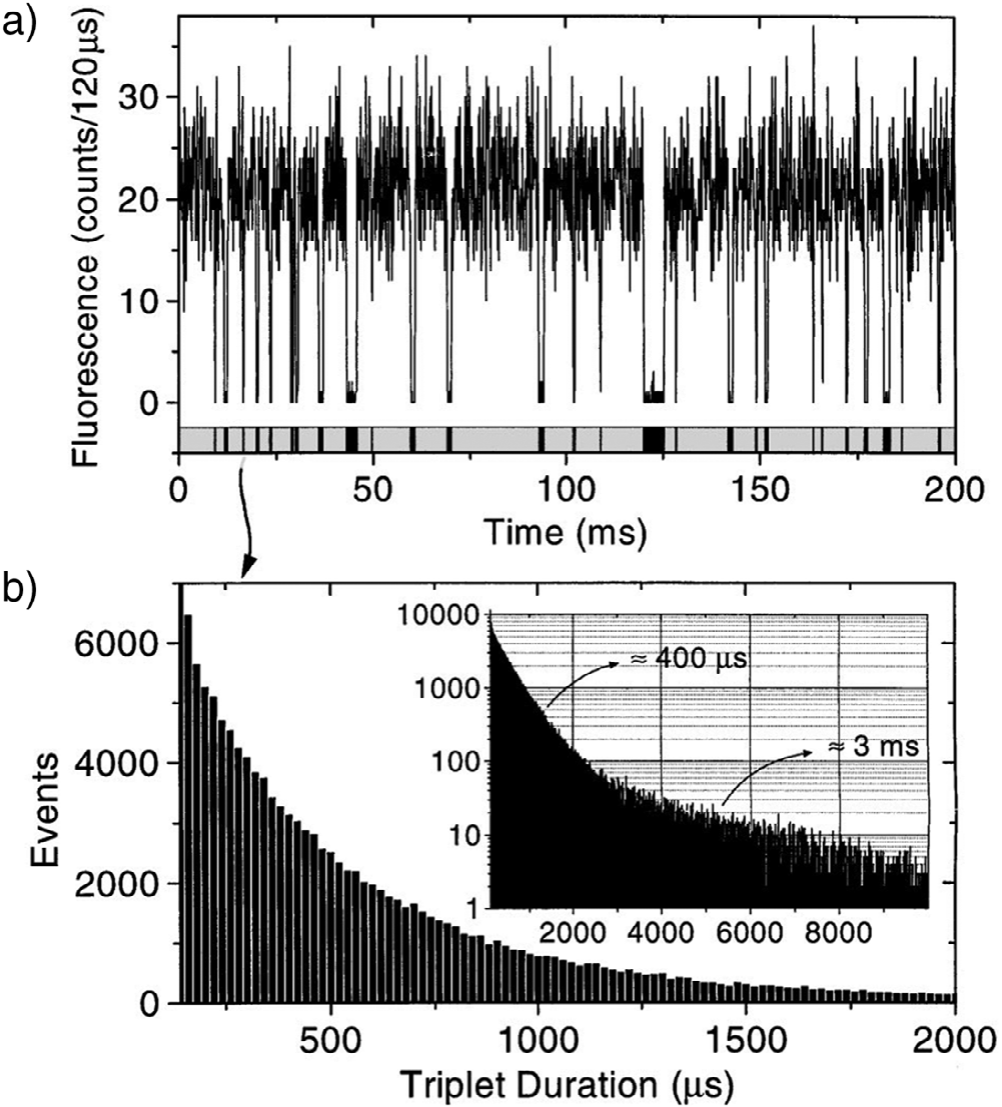
a) Part of a fluorescence time trace showing blinking obtained for a single terrylene molecule. b) Histogram of the extracted dark‐period durations. Inset: A semilog plot of the histogram reveals the different time constants in the triplet decay. Reproduced with permission from ref.^[^ ^41^ ^]^

Bypassing some of the stringent conditions, Brouwer et al. widened the scope of single‐molecule ODMR. They managed to measure ODMR signals on a single terrylene molecule,^[^ ^41^ ^]^ for which no ODMR signal can be detected with the conventional ODMR approach because of the unfavorable population and depopulation rates of the triplet sublevels. Their novel approach relied on measuring the durations of the individual dark periods, interrupting the periods of fluorescence, instead of the average fluorescence signal, see Figure 5a.

This requires a molecule that gives rise to strong fluorescence signals, as is the case for terrylene due to its low ISC probability in the triplet state (10^−5^) and its short fluorescence lifetime (3.8 ns).^[^ ^41^ ^]^ By constructing a histogram of the length of the triplet‐residence times, the decay of the triplet state can be determined, see Figure 5b. The ESR signal can be obtained by measuring the triplet decay for different microwave frequencies.

## 5 ODMR Using NV Centers

### 5.1 Fluorescence of a Single NV Center

**Figure 6:**
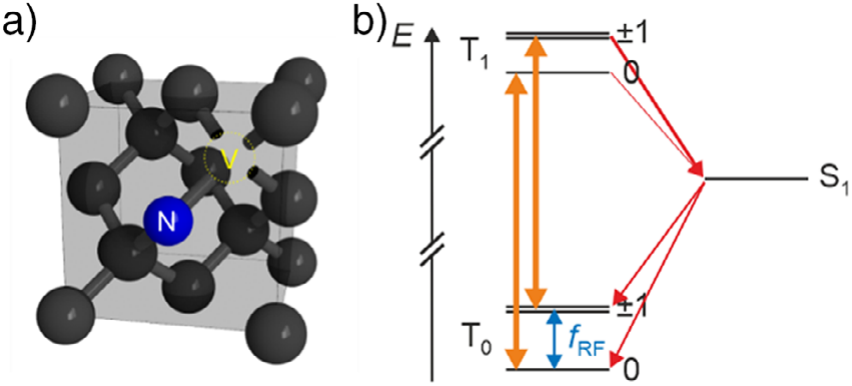
a) Lattice representation of a nitrogen‐vacancy (NV) center in the diamond lattice. b) Energy level scheme to measure ESR signals for a (single) NV center. The arrows are colored analogously to Figure 4a. There are multiple singlet states involved,^[^ ^44^ ^]^ here simplified as one. Conceptually based on a figure from Ref. [43].

To widen the scope of compounds that can be measured using ODMR, instead of measuring the molecules directly, NV centers can be employed as quantum sensors.^[^ ^42^ ^]^ An NV center is a point defect in diamond consisting of a substitutional nitrogen adjacent to a vacancy, as illustrated in Figure 6a. The negatively charged NV center has very favorable properties for ODMR such that signals can be measured for single NV centers.

In contrast to pentacene and terrylene, the NV center has a triplet ground state. It can be optically excited to its triplet excited state, and the luminescence of its decay into the ground state is measured. Because of the spin‐selective ISC into the singlet states, a strong nonequilibrium spin‐state population of the triplet states is induced, with the population of *m_S_* = 0 being largest. This is schematically depicted in Figure 6b. The probability for ISC into the singlet states is much larger for the *m_S_* = ±1 states than the one for the *m*_S_ = 0 state, whereas the ISC from the singlet states to the triplet ground state is much less spin selective.^[^ ^43^ ^]^ Since the transitions between the triplet sublevels are spin‐conserving, the ISC leads to a strong depletion of the *m_S_* = ±1 states. ESR can transfer population from *m_S_* = 0 to *m_S_* = ±1, which will preferentially end up in the dark singlet state in the following luminescence cycles because of the spin‐selective ISC rates mentioned above. Here, trapping in the singlet state leads to a reduction of the triplet‐to‐triplet luminescence, which is being detected. As this technique is also based on ODMR, many considerations are analogous to single‐molecule ODMR.

### 5.2 NV Center as a Quantum Sensor

An NV center can be used as a sensor to measure ESR signals on individual molecules, as was demonstrated for a protein,^[^ ^45^ ^]^ a DNA strand,^[^ ^46^ ^]^ and an endofullerene.^[^ ^47^ ^]^

To this end, double resonance spectroscopy is used, such as the DEER pulse sequence described at the end of section 2.

A particular strength of the method is its applicability at room temperature. In addition, the NV centers inside a nano‐sized diamond crystal are structurally stable and work robustly in different environments,^[^ ^48^ ^]^ enabling operation under ambient conditions and even in solution. This unique feature among the single‐molecule ESR techniques renders it ideal for biosensing and medical applications requiring physiological conditions.

**Figure 7:**
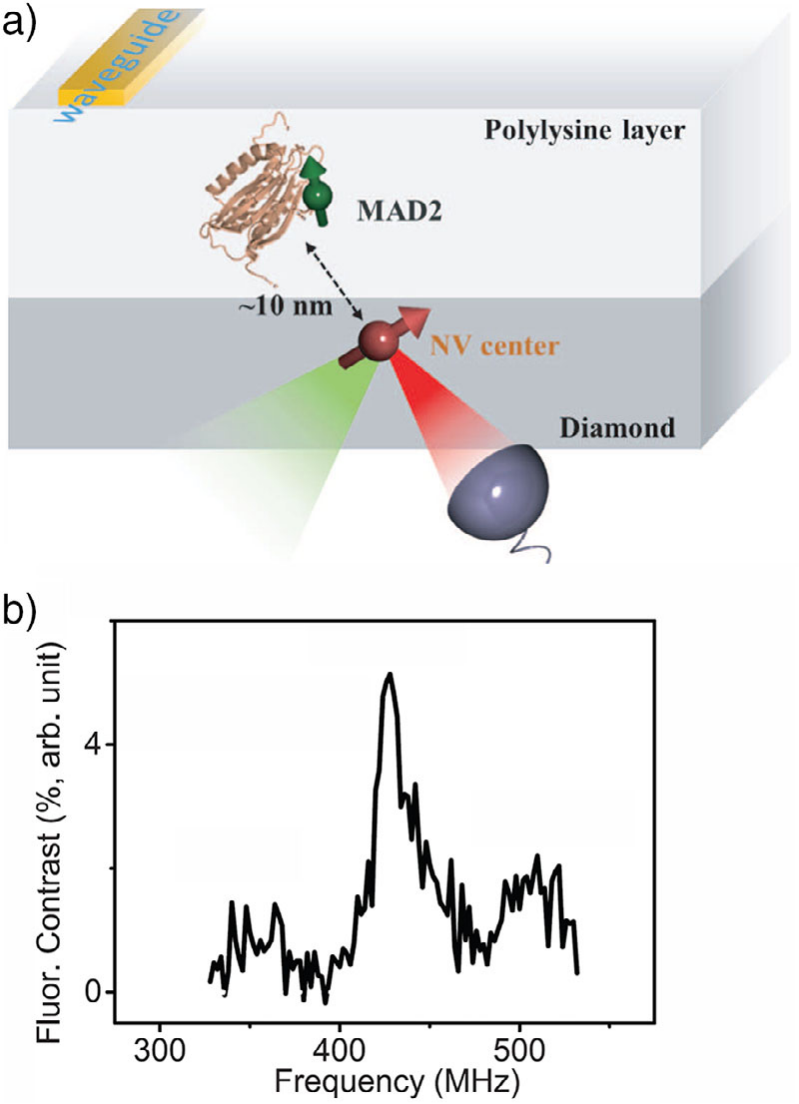
a) Schematic of the experimental setup used to detect a single mitotic arrest deficient‐2 (MAD2) protein using an NV center. An RF magnetic field is generated by a coplanar waveguide. The NV center's spin is optically addressed and read‐out. b) ESR spectrum of a nitroxide spin label of a MAD2 protein measured via an NV center. Reproduced and adapted with permission from ref. [45].

This method can only be applied to molecules with unpaired electron spins. Depending on the application, closed‐shell molecules can be functionalized with a spin‐carrying group, such as a nitroxide spin‐label. The NV center is only sensitive to molecules in its proximity. Molecules to be detected can either be embedded in a layer onto the diamond surface, such as a polylysine layer^[^ ^45^ ^]^ (see Figure 7a) or a network of molecules,^[^ ^47^ ^]^ or they can be covalently attached to the diamond surface.^[^ ^46^ ^]^ Thanks to the locality of the sensing, at a low coverage, the ESR signals measured in such DEER experiments were predominantly those of single molecules.

The spectra carry abundant additional information beyond structural information of the ESR‐active moiety itself. For example, Shi et al. proposed that the dynamics of the protein on the millisecond timescale are encoded in the broadening of the ESR signal's side peaks via a varying hyperfine coupling of the nitroxide spin label,^[^ ^45^ ^]^ see Figure 7b. Similarly, it was proposed that also intermolecular interactions in close‐to‐physiological environments can be inferred.^[^ ^46^ ^]^

## 6 Scanning‐Tunneling‐Based ESR

### 6.1 Measurement Principles of ESR‐STM

**Figure 8:**
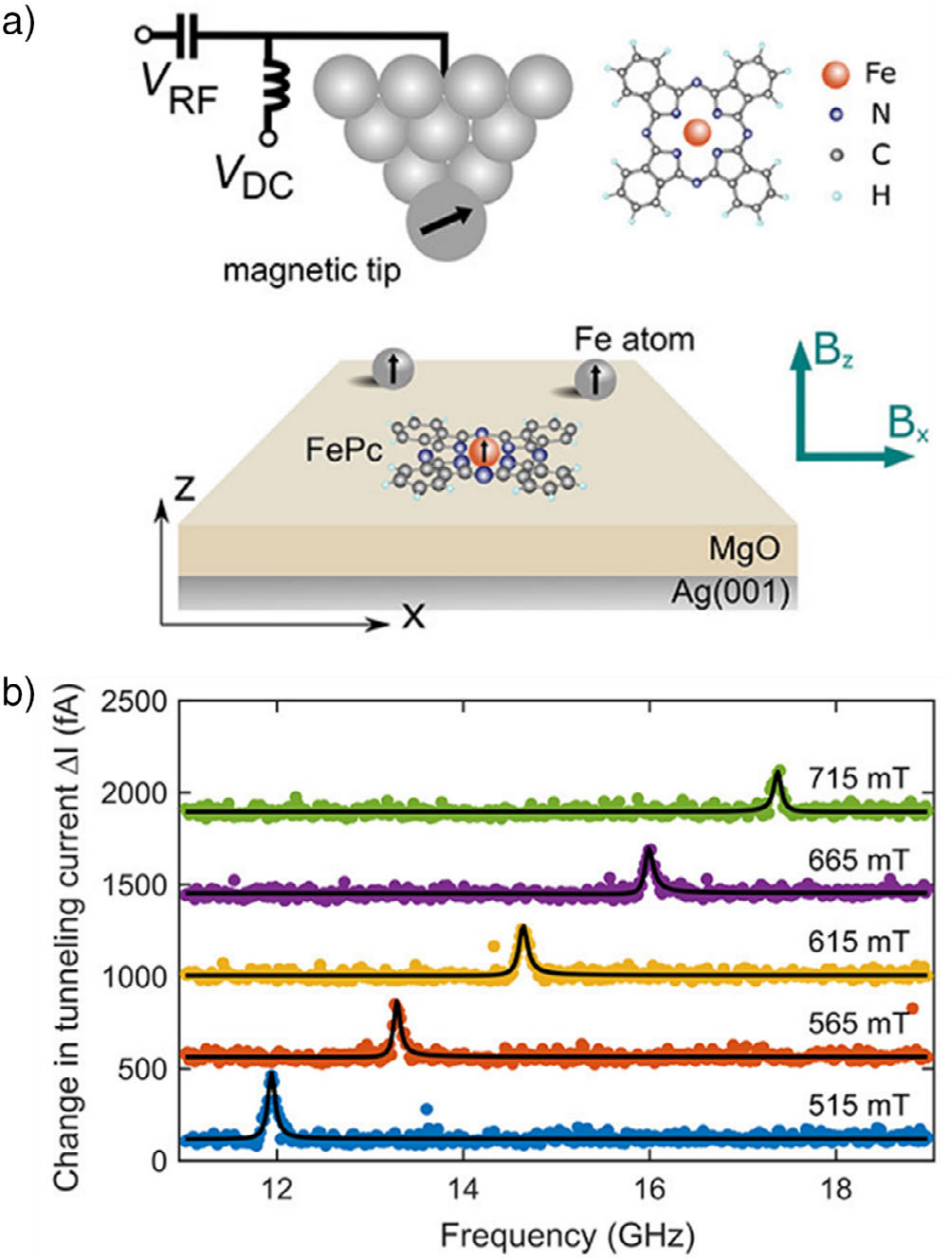
a) Schematic of the experimental setup to measure ESR on a single FePc molecule using STM. An effective RF magnetic field is generated indirectly by applying an RF voltage to the junction of magnetic tip and sample. b) ESR‐STM signals measured for a single FePc molecule at different magnetic fields, as indicated. Panels taken from Ref. [62]; license CC‐BY‐NC‐ND 4.0.

STM, as an atom‐resolving technique, is intrinsically single‐molecule sensitive. It measures the tunneling of electrons between an atomically sharp probe, the tip, and a conductive sample, see Figure 8a. Since magnetic resonance signals have been detected via electron transport,^[^ ^11^, ^12^, ^13^, ^14^, ^15^, ^24^ ^]^ it seems natural to perform atom‐resolving ESR by means of STM.

In fact, already in 1989 a current signal arising from the precession of individual paramagnetic spins has been reported,^[^ ^49^ ^]^ which was later also reported for single molecular spins.^[^ ^50^ ^]^ In these cases, the spin was not driven resonantly, but instead the spin precession was observed in the power spectrum at the resonance frequency. More than 25 years later, ESR‐STM with sub‐Ångström spatial resolution has been systematically demonstrated for individual atoms^[^ ^25^ ^]^ and molecules.^[^ ^51^, ^52^, ^53^, ^54^, ^55^ ^]^ Let us analyze the method of ESR‐STM along the four requirements introduced in section 2.1.

For spin‐1/2 molecules, non‐degenerate spin states result from the Zeeman effect by applying a magnetic field (requirement i). In most cases the population imbalance is given thermally at low temperatures according to the Boltzmann distribution (requirement iii). Whereas for ensemble measurements tiny differences in population suffice, for single‐spin measurements a large population imbalance becomes more important, such that many ESR‐STM experiments are conducted at a few‐Kelvin temperature (or even below 1 K) and a few‐Tesla field. From conventional magnetic resonance it is well known that there are several ways to achieve a hyperpolarization, that is, an imbalance in population far from thermal equilibrium, as is commonly used for nuclear spin systems.^[^ ^56^ ^]^ An electron‐spin hyperpolarization in ESR‐STM can be achieved by spin injection from the tip or sample, also referred to as spin‐transfer torque.

The resonant spin driving (requirement ii) requires an RF magnetic field. Yet, so far ESR‐STM used an RF *electric* field to drive the spin transitions. In an STM the applied bias voltage creates a huge electric field in the junction reaching atomic‐strength levels (≈ 10^9^ V m^−1^), such that adding an RF component to the bias represents a convenient way to implement RF driving (c.f. Figure 8a). Whereas the very details are still under discussion, it seems established that the atomically strong electric field modulates the coupling between the spin and the spin‐polarized tip^[^ ^57^, ^58^ ^]^ translating the RF *electric* field into an effective RF *magnetic* field.^[^ ^59^, ^60^ ^]^

Finally, the detection mechanism in ESR‐STM relies on spin polarized tunneling^[^ ^61^ ^]^ (see Figure 8b) (requirement iv). Because the spin is preserved upon tunneling, the tunneling rate depends on the relative alignment of the magnetization of tip and sample. Both spin components, along and perpendicular to the static field, lead to signatures in the current: The spin‐component along the static field does not oscillate at the Larmor frequency but simply changes with a spin flip, which is driven by the ESR resonance condition. Thus, the RF field can change the average spin‐component along the static field and hence – via spin‐polarized tunneling – the average current. In contrast, the spin‐component perpendicular to the static field oscillates at the Larmor frequency. Spin‐polarized tunneling leads thereby to an RF component in the current, which is usually not being detected because of the limited bandwidth in most STM experiments. However, in ESR‐STM at resonance conditions the bias voltage is also modulated at the same RF frequency (see above). The current is thus modulated in two ways at the same RF frequency, leading to a DC component of the current – an effect of frequency mixing in homodyne detection.

In one of the recent experiments on pentacene molecules yet another detection scheme has been exploited.^[^ ^53^ ^]^ Kovarik et al. applied a bias voltage at which the electrons sequentially tunneled via the singly occupied molecular orbital (SOMO) of the molecule. In a greatly simplified picture, the spin‐polarized tunneling electrons carry their magnetization to the SOMO. In magnetoelectronics this effect is commonly referred to as spin‐transfer torque.

The above leads to the following additional ESR‐STM detection mechanism^[^ ^53^ ^]^: The RF bias‐voltage modulation leads to an RF‐modulated current, leading to an RF‐modulated spin‐transfer torque, resulting in an RF‐modulated magnetization of the spin under investigation. Its component perpendicular to the static *B* field precesses at the resonance frequency, giving rise to two sources of current modulation at the same frequency and therefore to a DC component in the current due to homodyne frequency mixing, mentioned above.

### 6.2 ESR‐STM on Single Molecules

In pioneering works ESR‐STM has been applied to various organic and organometallic molecules, including iron phtalocyanine (FePc),^[^ ^51^, ^62^, ^63^ ^]^ bis(phthalocyaninato)Tb(III) (TbPc_2_),^[^ ^52^ ^]^ pentacene,^[^ ^53^ ^]^ perylenetetracarboxylic dianhydride (PTCDA),^[^ ^54^ ^]^ 4,5‐diaza‐9‐fluorenone (DAF),^[^ ^55^ ^]^ 9‐fluorenone,^[^ ^55^ ^]^ and 2,7‐dibromo‐9‐fluorenone (DBF).^[^ ^55^ ^]^

For adsorption of an atom or molecule directly on a metal surface the spin lifetime is typically too short for an ESR experiment. Most ESR‐STM measurements were performed for molecules adsorbed onto a bilayer of MgO, electronically decoupling the molecules from the required conductive substrate, commonly Ag(100).

The adsorption on a substrate opens a route to obtain a paramagnetic radical for closed‐shell molecules: because of the low work function of the MgO/Ag(100) surface, most adsorbed molecules accept an electron from the substrate and become negatively charged. Transitions of the unpaired electron spin (*S* = 1/2) can then be driven by the applied RF field. By varying the work function of the surface, molecules in different charge states can be measured. For example, ESR‐STM of neutral TbPc_2_ molecules could be measured using a NaCl/Cu(100) substrate.^[^ ^52^ ^]^

Maybe the most intriguing features of ESR‐STM are that ESR measurements can be combined with the atomic‐scale spatial resolution of STM, as well as its capability to manipulate single atoms and molecules. Thereby, the influence of the atomic environment on the spin properties can be investigated. For instance, the anisotropy of the exchange interaction between pairs of FePc molecules was quantified directly at atomic length scales. Its dependence on ligand orientation suggests a superexchange mechanism.^[^ ^51^ ^]^ For the same molecule, FePc, it was also found that the *T*_2_ relaxation time decreased with the increasing number of neighboring molecules, as attributed to their spin flipping.^[^ ^62^ ^]^ This pioneering work demonstrates how by means of ESR‐STM decoherence can be directly linked to the specific atomic‐scale environment. Finally, FePc─Fe complexes were formed by atomic manipulation and the complexes’ ESR signatures were investigated in detail, deviating profoundly from the ones of an isolated FePc.^[^ ^63^ ^]^

**Figure 9:**
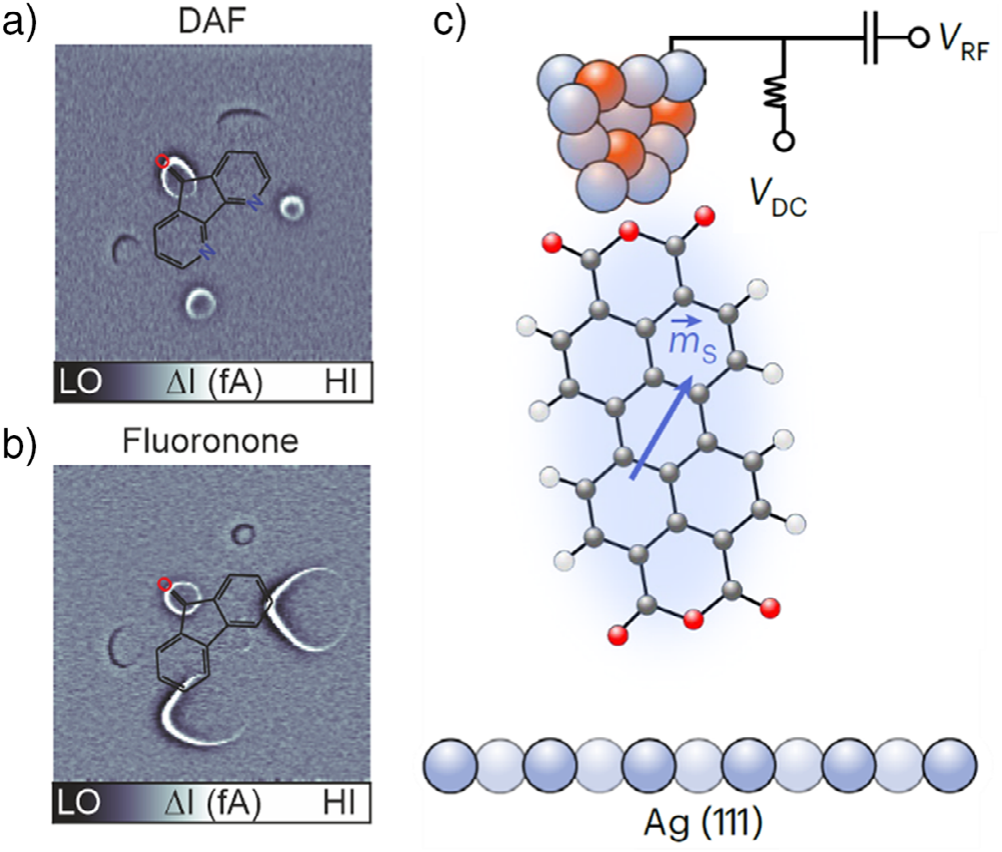
a), b) Magnetic‐resonance images on DAF and fluorenone molecules, respectively, measured using ESR‐STM. Adapted with permission from Ref. [55]. c) Experimental setup for using a PTCDA molecule as an ESR sensor on an STM tip. Adapted from Ref. [54]; license CC BY 4.0.

In addition, the magnetic STM tip can be used to spatially map the orbitals hosting the unpaired spin,^[^ ^64^ ^]^ which was used to perform magnetic resonance imaging (MRI) of single molecules^[^ ^55^, ^56^ ^]^ at sub‐molecular length scales. This type of MRI exploits the tip magnetic field to bring the spin into resonance. The locality of this effect allows visualizing the spin density, as demonstrated for single DAF and fluoronone molecules in Figure 9a,b. Although these molecules are structurally similar, MRI revealed pronounced differences.^[^ ^55^ ^]^

Finally, by transferring a single molecule to the STM tip, a single‐molecule ESR‐based quantum sensor can be created, which can be scanned over a variety of surfaces. Thereby, local electric and magnetic fields of the surface can be sensed with sub‐Ångström resolution. This has been demonstrated for a single PTCDA molecule as a sensor, which was attached to a spin‐polarized STM tip, as illustrated in Figure 9c.^[^ ^54^ ^]^ The binding configuration of the PTCDA molecule allowed the decoupling of its spin from the metallic tip, without the requirement of an insulating layer. The ESR‐STM resonance frequency of this PTCDA molecule depended on the local magnetic as well as electric fields at the sensor position above the surface. This way, the electric dipole moment of a Ag_2_ dimer and an Fe atom could be determined, as well as the magnetic moment of the latter.^[^ ^54^ ^]^

## 7 Atomic‐Force‐Based ESR

In contrast to STM, AFM does not require a conductive substrate. AFM‐based single‐molecule ESR therefore opens the prospect of investigating single‐molecule spin systems that are electronically decoupled from a substrate, expanding the scope of systems that can be studied and important for aiming at long spin‐coherence times. As in AFM forces are sensed,^[^ ^65^ ^]^ a detection mechanism exploiting dipole,^[^ ^26^ ^]^ or exchange^[^ ^66^ ^]^ forces to detect the spin by means of AFM seems natural and would open new research avenues in single‐molecule ESR, but has not yet been demonstrated. Alternatively, spin‐to‐charge conversion^[^ ^11^, ^12^, ^13^, ^14^, ^15^ ^]^ can be used to sense the spin state via the single‐charge sensitivity of AFM, as explained here.

### 7.1 Measuring Triplet Lifetime with AFM

A key methodological foundation to the realization of ESR‐AFM was the detection of the triplet‐state lifetime of individual pentacene molecules by means of AFM,^[^ ^67^ ^]^ with the submolecular imaging capability of AFM being available. The triplet‐state lifetime changes upon driving ESR transitions between the zero‐field triplet sublevels and therefore enables to perform single‐molecule ESR via lifetime detection similarly as in ODMR.

**Figure 10:**
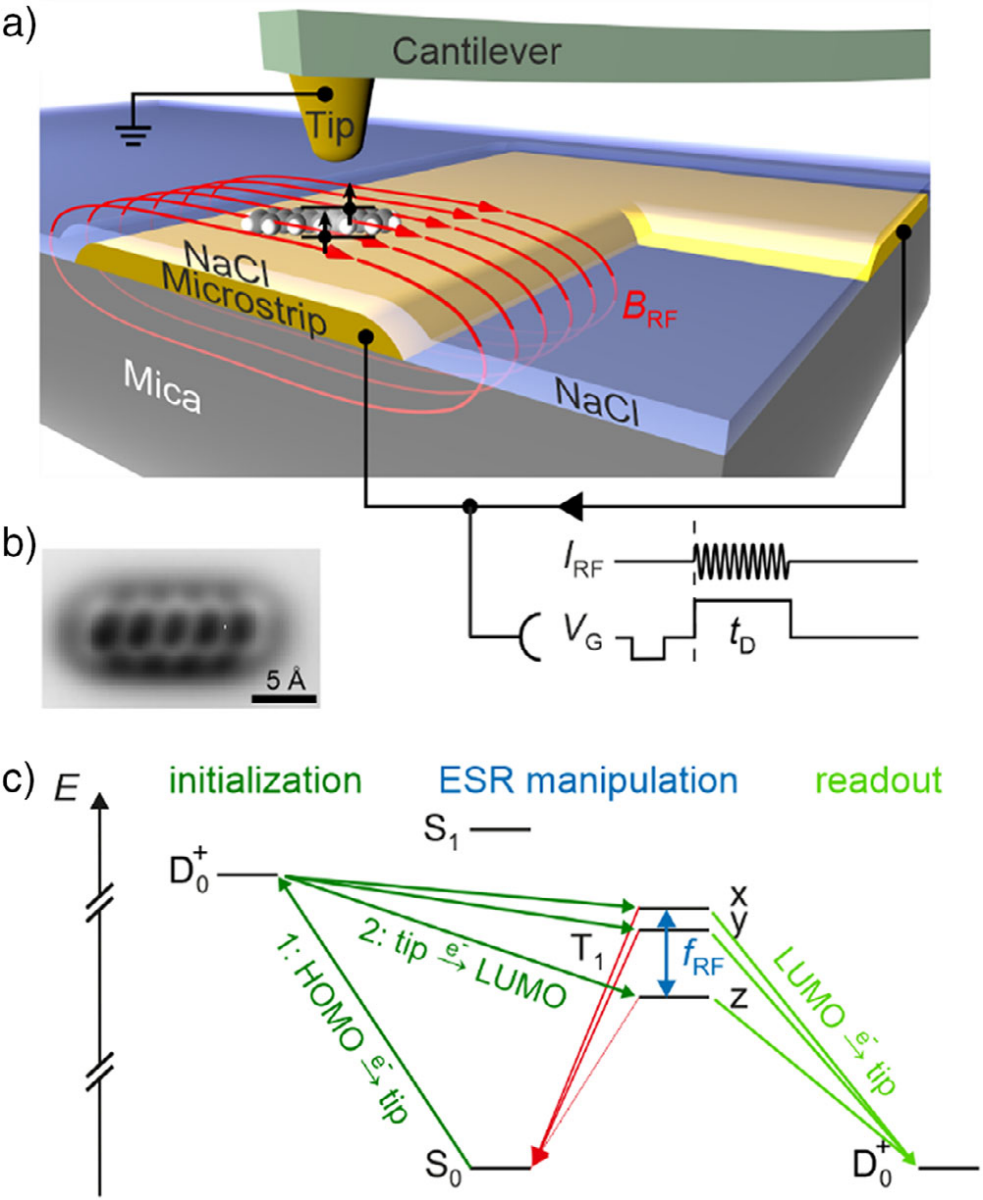
a) Schematic of the experimental setup to measure ESR of a single pentacene molecule using AFM. An RF magnetic field is generated by sending an RF current through the microstrip. Voltage pulses are applied to steer electron tunneling between the AFM tip and the molecule. b) AFM image of a single pentacene molecule. c) Level scheme of pentacene with the transitions used for ESR‐AFM: the molecule is initialized in two subsequent voltage‐controlled tunneling events between tip and molecule (dark green). The RF driving and decay is analogous to ODMR shown in Figure 4, but the readout proceeds via spin‐to‐charge conversion by tunneling transitions to the tip (light green). The level alignment of the positively charged doublet D_0_^+^ depends on the voltage and is therefore displayed twice: On the left hand side for the voltage applied during the initialization and on the right hand side for the voltage applied during readout. Panels a and b adapted from Ref. [16]; license CC BY 4.0.

The triplet‐lifetime measurements were performed on molecules adsorbed on an insulating film that electronically decouples the molecules from a conducting surface, see Figure 10a. In a single‐molecule experiment, the measurement of a lifetime has to proceed in repeated cycles. By applying cyclic sequences of voltage pulses to the underlying conducting sample support, electron tunneling can be steered between the conductive AFM tip and the molecule. Two subsequent controlled tunneling events bring the molecule to its triplet state (dark green arrows in Figure 10b). After a controlled dwell time, the remaining triplet population is read‐out by a spin‐to‐charge conversion^[^ ^11^, ^12^, ^13^, ^14^, ^15^, ^67^ ^]^: the population in the triplet state is mapped onto the positive charge state (light green arrows in Figure 10b), while the population in the singlet ground state remains in this state. These two different charge states can be discriminated in the AFM signal during the read‐out period. By varying the duration of the dwell time, the triplet lifetime can be measured.^[^ ^67^ ^]^

### 7.2 ESR‐AFM

To measure ESR‐AFM spectra,^[^ ^16^ ^]^ an RF magnetic field was applied via a microstrip (see Figure 10a) and the RF frequency was swept with fixed dwell time. If the frequency of the RF magnetic field is in resonance with the splitting between two triplet sublevels, the triplet lifetime will be reduced, which was detected as a reduction of the measured triplet population – similar as in ODMR. Hence, although the detection mechanism (iv) is clearly different from ODMR, concerning the first three requirements (i‐iii), ESR‐AFM works quite analogously to ODMR. Thereby, the spin‐state population is not given thermally and therefore does not rely on low temperatures – although working at low temperatures is often desired for other technical reasons, such as to stabilize individual molecules on the surface.

**Figure 11:**
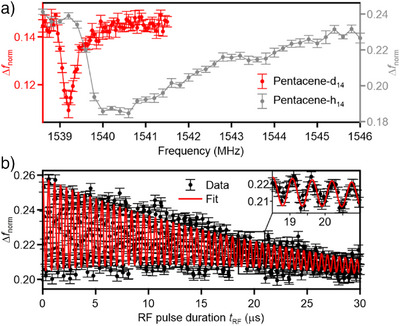
a) ESR‐AFM spectra of single pentacene‐d_14_ and pentacene‐h_14_ molecules as indicated. b) Rabi oscillations measured for a single pentacene‐d_14_ molecule using ESR‐AFM. Adapted from Ref. [16]; license CC BY 4.0.

ESR‐AFM combines ESR with the atomic‐scale spatial resolution offered by AFM. Thereby, locally different isotopomers of pentacene could be discriminated (see Figure 11a) and the ESR selection rules could be demonstrated in real space.^[^ ^16^ ^]^

In addition, Rabi oscillations were measured for protonated and deuterated pentacene molecules. ESR‐AFM allowed the observation of Rabi oscillations over tens of microseconds^[^ ^16^ ^]^ (see Figure 11b) highlighting the role of electronic decoupling in comparison to ESR‐STM.

So far, ESR‐AFM has only been applied to pentacene and PTCDA. For this implementation of ESR‐AFM, molecules have to meet the following requirements. First, the molecule needs to be stably adsorbed onto an insulating film in multiple charge states and under the application of voltage pulses of a few volts. Second, at least two of the three triplet sublevels should have sufficiently different lifetimes (roughly at least by a factor of two). Third, the fundamental gap of the molecule (difference between ionization potential and electron affinity) needs to be larger than twice the S_0_‐T_1_ energy difference.^[^ ^68^ ^]^

The applicability of AFM based ESR could be extended by fundamentally different ESR‐AFM measurement schemes that for instance rely on the detection of dipolar or exchange forces.

## 8 Conclusion and Outlook

Single‐molecule ESR can reveal information that would be concealed in an ensemble average. For example, if a molecule can be found in many different configurations, structure analysis by ESR can now be done in a one‐by‐one fashion. Similarly, to understand the effect that the environment has on a spin system – including the microscopic origins of magnetic and electrical noise, as well as disorder – single‐molecule experiments are crucial. ODMR has laid the foundation for single‐molecule ESR. While NV‐center‐based single‐molecule ESR is also based on ODMR, it adds a complementary aspect: the spin state of the molecule is sensed via the NV center. NV‐center based single‐molecule ESR, thereby, expanded the applicability of ODMR to a wide range of molecules, including biologically relevant ones. This technique has also the unique feature to be compatible to ambient and even physiological conditions, such that medical applications are within reach.

In contrast to the ODMR approaches, the two scanning‐probe‐based single‐molecule ESR techniques, ESR‐STM and ESR‐AFM offer Ångström‐scale spatial resolution as well as atom and molecular manipulation.^[^ ^69^, ^70^, ^71^ ^]^ The high spatial resolution can be used to map out spin densities within a single molecule as has already been demonstrated.^[^ ^56^, ^62^ ^]^ In MRI, hyperfine interaction might allow for individual isotope localization within molecules and isotope tracking in on‐surface chemical reactions in the future. Atomic‐scale imaging can also be used to correlate molecular configurations or the atomic‐scale environment with the ESR signal. Combined with atomic and molecular manipulation techniques, single‐molecule ESR may pave the way for quantum computing in artificial atomically precise spin structures.^[^ ^72^ ^]^ The complementarity of ESR‐STM and ESR‐AFM in comparison to ODMR is underlined by different experimental conditions. For example, the scanning‐probe techniques require stable adsorption of the target molecule on a surface under ultra‐high vacuum, while ODMR allows studying molecules with a three‐dimensional structure, molecules embedded in solid matrices and even under ambient conditions. Prior works based on ESR‐STM and ESR‐AFM also show a large degree of complementarity between the two: At present, ESR‐STM operates much faster, making it ideal for MRI. ESR‐STM has also been combined with atomic and molecular manipulation, whereas ESR‐AFM demonstrated longer coherence times and reduced line broadening.

## Conflict of Interests

The authors declare no conflict of interest.

## Data Availability

Data sharing is not applicable to this article as no new data were created or analyzed in this study.
